# Association between urinary sodium and circulating lipid levels: a Mendelian randomization study

**DOI:** 10.3389/fendo.2023.1189473

**Published:** 2023-11-29

**Authors:** Chi Yuan, Peijia Jing, Zhongyu Jian, Xin Wei

**Affiliations:** ^1^Department of Pediatric Surgery, West China Hospital, Sichuan University, Chengdu, China; ^2^Department of Urology, Institute of Urology (Laboratory of Reconstructive Urology), West China Hospital, Sichuan University, Chengdu, China; ^3^Department of Rehabilitation Medicine, West China Hospital, Sichuan University, Chengdu, China; ^4^West China Biomedical Big Data Center, Sichuan University, Chengdu, China

**Keywords:** circulating lipid levels, urinary sodium, Mendelian randomization, HDL-C, LDL- C

## Abstract

**Background:**

Urinary sodium was indicated to be associated with dyslipidemia, but inconsistent conclusions for this association exist across the present observational studies.

**Objectives:**

This study aimed to evaluate the causal association between urinary sodium and circulating lipid levels [low-density lipoprotein cholesterol (LDL-C), triglycerides, and high-density lipoprotein cholesterol (HDL-C)] through Mendelian randomization.

**Methods:**

Univariable Mendelian randomization (UVMR) and multivariable Mendelian randomization (MVMR) with pleiotropy-resistant methods were performed. Data for urinary sodium were obtained from the genome-wide association study (GWAS) from 446,237 European individuals. Data for lipid profiles were extracted from GWAS based on the UK Biobank (for the discovery analysis) and the Global Lipids Genetics Consortium (for the replication analysis).

**Results:**

In the discovery analysis, UVMR provided evidence that per 1-unit log-transformed genetically increased urinary sodium was associated with a lower level of HDL-C level (beta = −0.32; 95% CI: −0.43, −0.20; *p* = 7.25E−08), but not with LDL-C and triglycerides. This effect was still significant in the further MVMR when considering the effect of BMI or the other two lipid contents. In contrast, higher genetically predicted triglycerides could increase urinary sodium in both UVMR (beta = 0.030; 95% CI: 0.020, −0.039; *p* = 2.12E−10) and MVMR analyses (beta = 0.029; 95% CI: 0.019, 0.037; *p* = 8.13E−10). Similar results between triglycerides and urinary sodium were found in the replication analysis.

**Conclusion:**

Increased urinary sodium may have weak causal effects on decreased circulating HDL-C levels. Furthermore, genetically higher triglyceride levels may have independent causal effects on increased urinary sodium excretion.

## Introduction

Dyslipidemia represents the imbalance of circulating lipid levels like low-density lipoprotein cholesterol (LDL-C), triglycerides, and high-density lipoprotein cholesterol (HDL-C), which leads to severe diseases in other organ systems, especially cardiovascular disease (CVD) (1). Since CVD is the leading cause of mortality in the world, the identification of causal risk factors associated with dyslipidemia would provide important insights into preventing CVD (2).

Over the past decades, high sodium consumption has been considered closely related to CVD by promoting the development of hypertension (3). As the golden standard for estimating salt intake, urinary sodium has been indicated and associated in a dose–response manner with a higher CVD risk (4). Unfortunately, few publications have estimated the association between urinary sodium and dyslipidemia based on observational studies. Due to the heterogeneity in the selection of cases, controls, sample size, and study designs, there were inconsistent conclusions across the present publications (5–8). Moreover, because of the potential confounding and reverse causation, observational studies are limited in estimating causal association.

A randomized control trial (RCT) has been long regarded as the golden standard for causality estimation, which can control the confounding factors and provide a causal estimate with a high evidence level. However, conducting a highly qualified RCT requires abundant time and resources. By applying genetic variants related to the exposure of interest as the instrumental variable (IV), Mendelian randomization (MR) can minimize unmeasured confounding from observational studies and estimate the causal association between potential risk factors and health outcomes (9). When an RCT is not easily practicable, a precisely designed MR can provide more reliable evidence to guide interventional research than observational ones and complementary information for further RCTs (10).

The aims of this study were to explore whether 1) urinary sodium exerts total and direct causal effects on circulating lipid levels (LDL-C, triglycerides, and HDL-C) and 2) circulating lipid levels have total and directional causal effects on the urinary sodium.

## Material and method

### Study design

We conducted this study with several steps of a two-sample MR. First, we applied bidirectional univariable MR (UVMR) to assess the causal association between urinary sodium and circulating lipid levels (the discovery analysis). Second, we used bidirectional multivariable MR (MVMR) to evaluate the direct effect of urinary sodium on circulating lipid levels and the direct effect of circulating lipid levels on urinary sodium when accounting for body mass index (BMI) (the discovery analysis). In each step above, we performed replication analysis with data of circulating lipid levels from another database (the Global Lipids Genetics Consortium). **Supplementary Figures 1, 2** show the flow graph of each procedure.

### Data source

#### Urinary sodium

The data on urinary sodium were obtained from 446,237 European individuals from the UK Biobank (UKB) (**Supplementary Table 1**) (11). The sodium concentration in collected urine samples was determined by the ion-selective electrode method (potentiometric method) using Beckman Coulter AU5400, UK Ltd., in which the analytic range for sodium was 2–200 mmol/L and the coefficients of variation (CV) of the low and high internal quality control (IQC) level of urinary sodium were 0.99% and 0.82%, respectively (12). Participants with sex discordance, high missingness/heterozygosity, and withdrawn consent, as well as those who were pregnant or unsure of their pregnancy status at baseline, were excluded. A custom Affymetrix UKB Axiom array was applied for the genotyping of DNA samples obtained from the UKB. The urinary sodium was log-transformed and obtained with a linear mixed model controlling for population stratification and correlation among individuals and was adjusted for age and sex.

#### Circulating lipid levels

The data of circulating lipid levels for the discovery analysis were obtained from a largest-to-date genome-wide association study (GWAS) with participants of European ancestry from the UKB (sample size: triglyceride: *n* = 441,016, HDL-C: *n* = 403,943, LDL-C: *n* = 440,546) (13). The lipid traits (unit: standard deviation [SD] [mmol/L]) were standardized/normalized using inverse rank normalization, and the analyses were adjusted for age, sex, and genotyping chip array. Details regarding sample handling and the assays employed have been previously elucidated in other publications (14, 15).

In summary, Beckman Coulter (UK), Ltd. provided assays using the Beckman Coulter AU5800 platform, and the methods included enzyme immuno-inhibition for HDL-C, enzymatic selective protection for LDL-C, and enzymatic for triglycerides. The CV for HDL-C at the low, medium, and high IQC levels were 1.81%, 1.76%, and 1.72%, respectively. For LDL-C, the CV was 1.71%, 1.59%, and 1.57% at the corresponding IQC levels, while for triglycerides, it was 2.27%, 2.18%, and 2.05%, respectively.

The data of circulating lipid levels for the replication analysis were extracted from the most representative GWAS of subjects from the Global Lipids Genetics Consortium (sample size: triglyceride: *n* = 177,861, HDL-C: *n* = 187,167, LDL-C: *n* = 173,082) (16). This study collected the summary statistics for Metabochip SNPs from 45 studies. Individuals who were known to be taking lipid-lowering medications were excluded. LDL-C levels were directly measured in 10 studies, representing 24% of the total study population, while in the remaining studies, they were estimated using the Friedewald formula (16, 17). The circulating lipid levels (unit: SD [mg/dL]) were measured after fasting for more than 8 h and adjusted for age, age^2^, and sex and then quantile-normalized. Quality control steps involved identifying outliers, ensuring consistent strand assignment, validating reported statistics, checking genomic control values, and excluding rare variants (16). This GWAS only selected European individuals for novel genome-wide significant loci discovery, while the non-European individuals were examined only for fine-mapping analyses.

### Statistical analyses and Mendelian randomization

We applied TwoSampleMR (https://github.com/MRCIEU/TwoSampleMR) to combine and harmonize data in UVMR, bidirectional MR, and MVMR. The random-effects inverse-variance weighted (IVW) method was utilized as the primary method to provide a robust causal estimate in the absence of directional pleiotropy. However, the IVW method could ignore the potential pleiotropy, which could lead to the violence of instrumental variable assumptions of MR (18). Therefore, we applied sensitivity analysis like MR-Egger, weighted median, and weighted mode methods (19). Furthermore, Cochran’s Q statistic was used for the detection of possible heterogeneities (*p*-value < 0.05 indicated the presence of heterogeneity). The potential horizontal pleiotropy was tested by intercept obtained from the MR-Egger analysis (*p*-value < 0.05 indicated the presence of horizontal pleiotropy). To exclude the single nucleotide polymorphism (SNP) explaining more variation for the outcome rather than the exposure, we applied Steiger filtering to reduce the possibility of false results because of pleiotropy and the MR Pleiotropy RESidual Sum and Outlier (MR-PRESSO) method for the detection and removal of potential outliers in IVW regression (20). Since the lipid contents could have reciprocal genetic effects on each other and obesity has been considered closely associated with lipid metabolism (21), we conducted MVMR to estimate the direct causal relationship between urinary sodium and circulating lipid levels by considering the effect of BMI and other two lipid contents.

### Mendelian randomization assumptions and results interpretation

The MR analysis should meet the following three assumptions to guarantee the robustness of results: 1) the IVs should robustly associate with the exposure, 2) the IVs cannot associate with confounders, and 3) the IVs should only affect the outcome through exposure (19). To meet assumption 1, we only selected genetic variants significantly associated with LDL-C and HDL-C, triglycerides, and urinary sodium (*p*-value < 1 × 10^−8^ for urinary sodium and *p*-value < 5 × 10^−8^ for circulating lipid level) as IVs. Moreover, the calculated *F*-statistics for exposures were all larger than 10 (**Supplementary Table 2**), which minimized the bias from weak instruments (22). Furthermore, to meet assumptions 2 and 3, we identified and excluded genetic variants that are in a state of linkage disequilibrium (LD) (*r*^2^ < 0.01, LD distance > 10,000kb) and performed sensitivity analyses (MR-Egger, weighted median, and weighted mode methods), Steiger filtering, and MR-PRESSO to diminish potential pleiotropic effects (19). Furthermore, we conducted Cochran’s Q statistic for the estimation of heterogeneity to further test assumptions 2 and 3 since the existence of heterogeneity may result in pleiotropy of SNPs.

The MR analyses were conducted in R version 4.1.1 with TwoSampleMR, MendelianRandomization, MR‐PRESSO, and MVMR R packages. All the *p*-values are two-tailed. When the *p*-value is less than 0.05 in the IVW method and the association direction remained consistent in the results of MR-Egger, weighted median, or weighted mode, the suggestive causal associations were considered. The causal effects were reported in beta coefficients since all the outcomes are continuous. The unit for urinary sodium and lipid traits was in the SD scale.

## Results

### UVMR: bidirectional relationship between urinary sodium and circulating lipid levels

**Figure 1:**
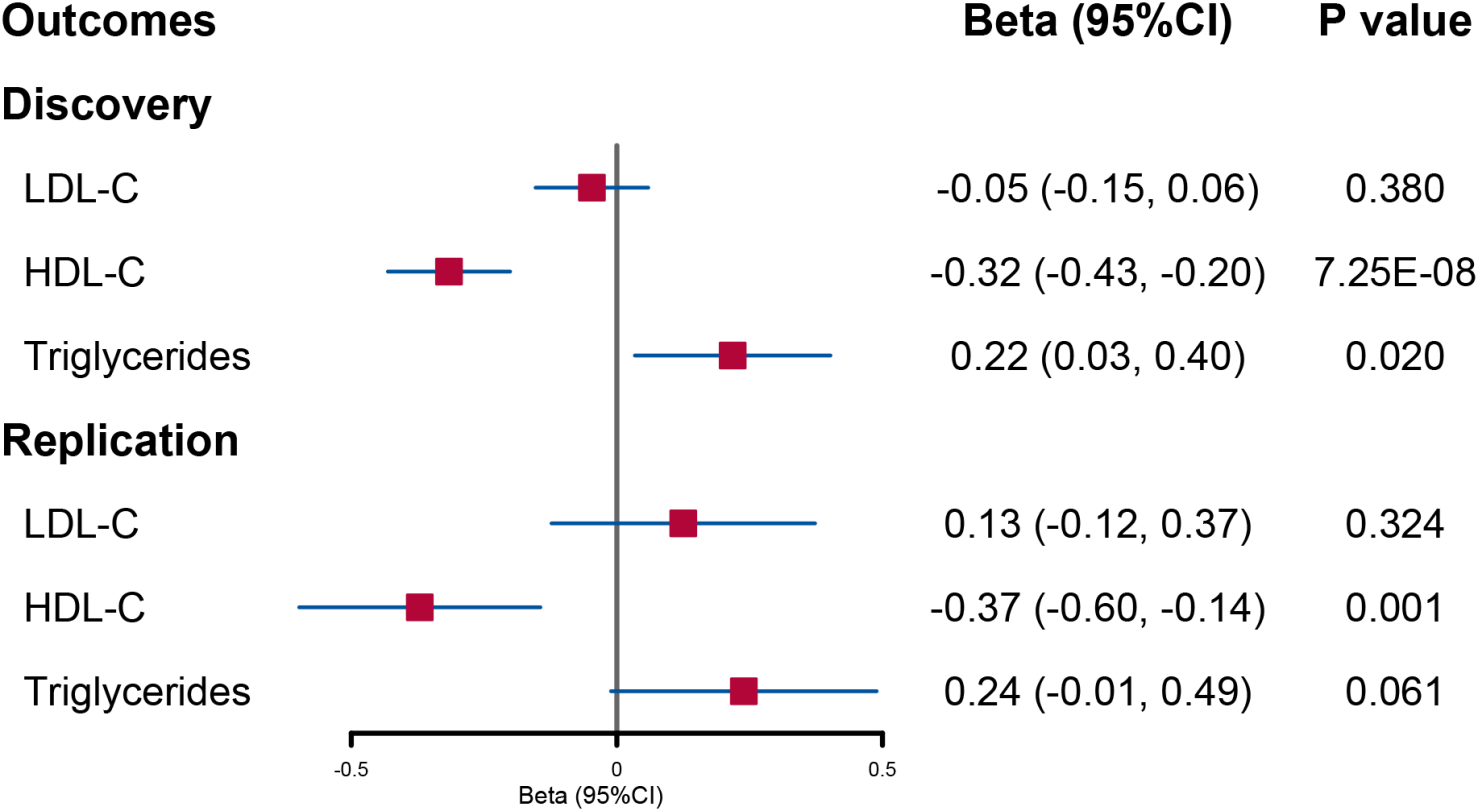
Forest plot of univariable Mendelian randomization analysis for the effect of urinary sodium on circulating lipid level. LDL-C, low-density lipoprotein cholesterol; HDL-C, high-density lipoprotein cholesterol.

In the discovery analysis, we initially included 48 SNPs for urinary sodium. After using the MR-PRESSO and Steiger filter method, there were 10, 16, and 12 SNPs excluded when LDL-C, HDL-C, and triglycerides were utilized as outcomes, respectively (**Supplementary Figure 1**). We observe that per 1-SD genetically increased log-transformed urinary sodium would reduce circulating HDL-C levels (beta = −0.32; 95% CI: −0.43, −0.20; *p* = 7.25E−08) but increase triglyceride levels (beta = 0.22; 95% CI: 0.03, 0.4; *p* = 0.02) (**Figure 1** and **Supplementary Table 3**). Consistent results in IVW estimates were obtained in weighted median and weighted mode methods (**Supplementary Table 3**). However, there was no evidence that the change in urinary sodium would affect circulating LDL-C levels (**Figure 1** and **Supplementary Table 3**).

**Figure 2:**
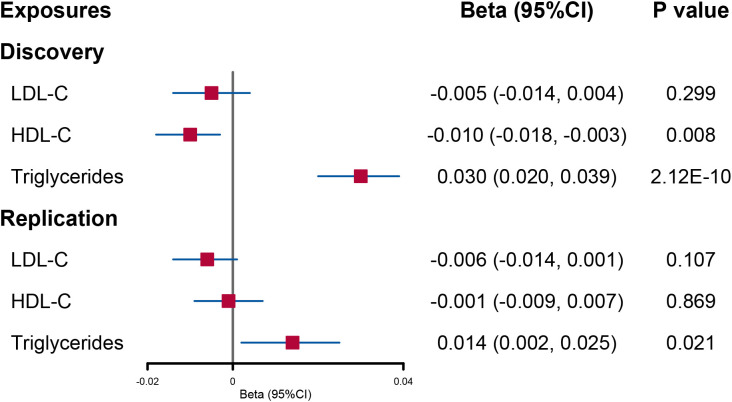
Forest plot of univariable Mendelian randomization analysis for the effect of circulating lipid level on urinary sodium. LDL-C, low-density lipoprotein cholesterol; HDL-C, high-density lipoprotein cholesterol.

When the lipid levels were utilized as exposures, there were 175 SNPs for LDL-C, 434 SNPs for HDL-C, and 374 SNPs for triglycerides included in the discovery analysis (**Supplementary Figure 2**). An attenuated urinary sodium level was observed when circulating HDL-C levels increased (beta = −0.010; 95% CI: −0.018, −0.003; *p* = 0.008). Reversely, increased triglyceride levels were indicated to elevate urinary sodium level (beta = 0.030; 95% CI: 0.020, −0.039; *p* = 2.12E−10) (**Figure 2** and **Supplementary Table 5**).

The *F*-statistic values are 45–46 for instruments in lipid traits (**Supplementary Table 2**). Heterogeneity was found between SNPs of urinary sodium and lipid level, but no evidence for the presence of horizontal pleiotropy was provided by the MR-Egger intercept (**Supplementary Table 3**).

### MVMR: bidirectional relationship between urinary sodium and circulating lipid levels

**Figure 3:**
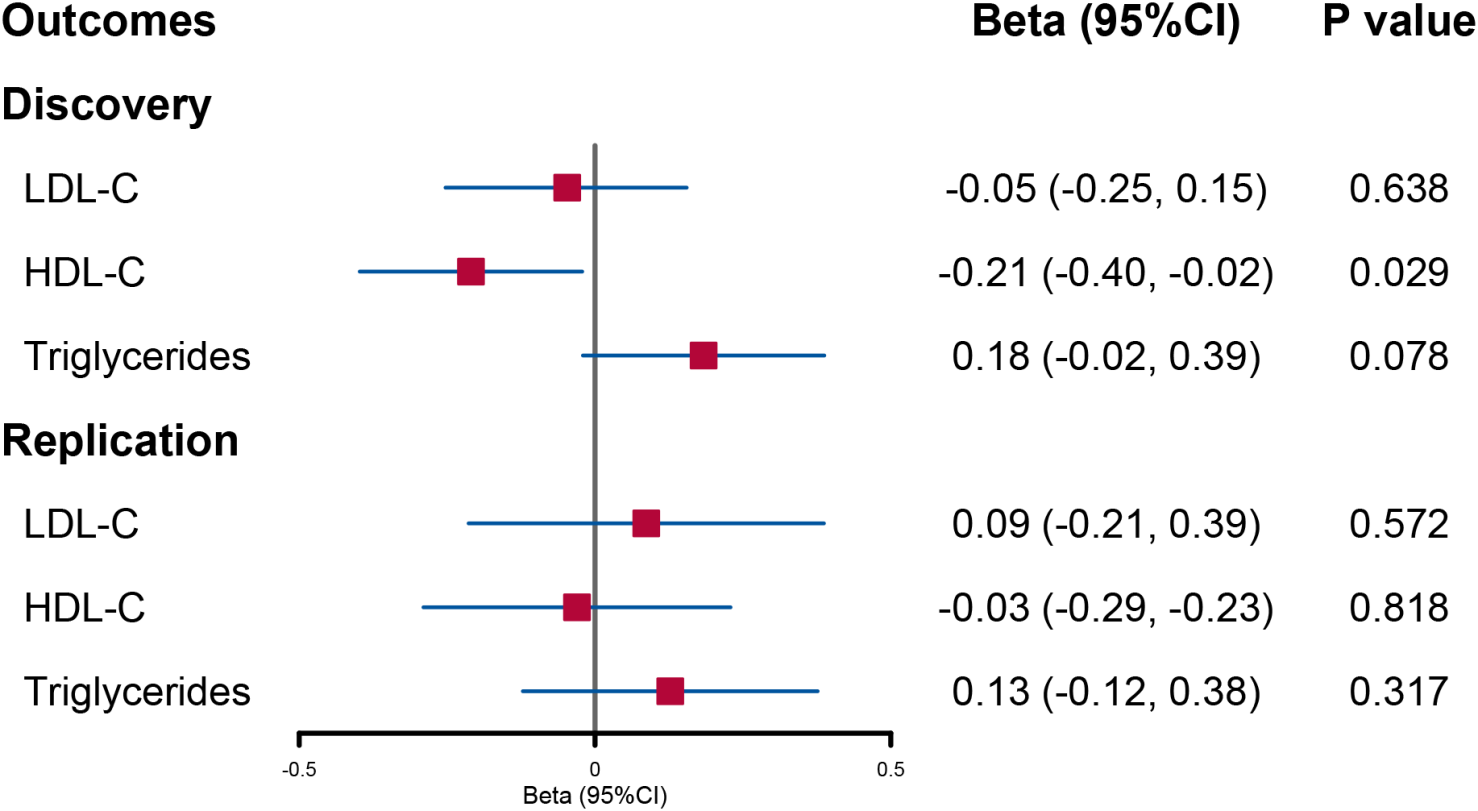
Forest plot of multivariable Mendelian randomization analysis for the effect of urinary sodium on circulating lipid level. LDL-C, low-density lipoprotein cholesterol; HDL-C, high-density lipoprotein cholesterol.

When accounting for the effect of BMI and the other two lipid contents, the decreased trend in circulating HDL-C level per 1-SD genetically increased log-transformed urinary sodium found in UVMR remained unchanged although the statistical power was weakened (beta = −0.21; 95% CI: −0.40, −0.02; *p* = 0.029). However, the direct causal effect of urinary sodium on triglycerides was not significant (**Figure 3**).

**Figure 4:**
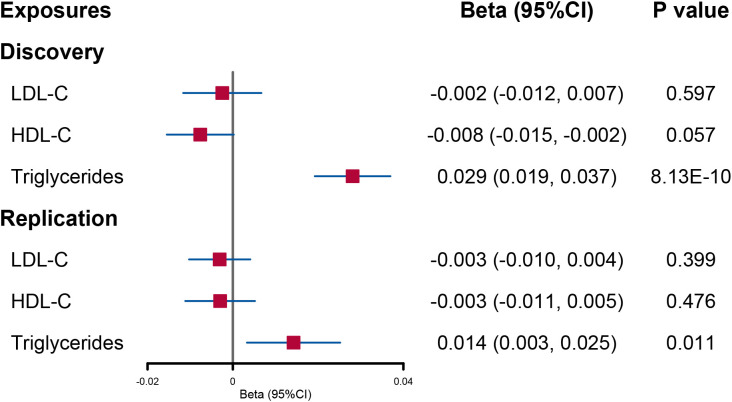
Forest plot of multivariable Mendelian randomization analysis for the effect of circulating lipid level on urinary sodium. LDL-C, low-density lipoprotein cholesterol; HDL-C, high-density lipoprotein cholesterol.

When lipid levels were utilized as exposures, there was evidence that increased triglyceride levels had a direct causal effect on urinary sodium (beta = 0.029; 95% CI: 0.019, 0.037; *p* = 8.13E−10). No significant causal effect of LDL-C and HDL-C on urinary sodium was found (**Figure 4**).

### Replication analysis

In the replication analysis, we initially included 28 SNPs for urinary sodium. After using the MR-PRESSO and Steiger filter method, there were 2, 2, and 8 SNPs excluded when LDL-C, HDL-C, and triglycerides were utilized as outcomes, respectively (**Supplementary Figure 1**).

In UVMR, a reduced HDL-C level per 1-SD increased urinary sodium remained significant although the statistical power was reduced (beta = −0.37; 95% CI: −0.60, −0.14; *p* = 0.001) (**Figure 1** and **Supplementary Table 4**). However, the causal effect of urinary sodium on triglyceride levels was weakened to null (beta = 0.24; 95% CI: −0.01, 0.49; *p* = 0.061) (**Figure 1** and **Supplementary Table 4**). When the lipid levels were utilized as exposures, there were 93 SNPs for LDL-C, 116 SNPs for HDL-C, and 65 SNPs for triglycerides included in the discovery analysis (**Supplementary Figure 2**). The effect of circulating HDL-C levels on urinary sodium level was not found to be significant (beta = −0.001; 95% CI: −0.009, 0.007; *p* = 0.869). On the contrary, a significant effect of higher triglyceride levels on increased urinary sodium level was found (beta = 0.014; 95% CI: 0.002, 0.025; *p* = 0.021) (**Figure 2** and **Supplementary Table 6**).

In the replication analysis of MVMR, no significant association was found when urinary sodium was utilized as the exposure (**Figure 3**). However, when lipids were utilized as outcomes, a direct causal effect of increased triglyceride levels on urinary sodium still holds (beta = 0.014; 95% CI: 0.003, 0.025; *p* = 0.011) (**Figure 4**).

## Discussion

In this genetics-based causal investigation, we reported genetic evidence for the bidirectional causal association between urinary sodium and circulating lipid levels. The results of UVMR supported that increased urinary sodium would decrease circulating HDL-C levels, but only a weak association was found in the further MVMR. Meanwhile, higher genetically predicted triglycerides could increase urinary sodium, and the tendency holds in both UVMR and MVMR analyses. Collectively, our results suggested the bidirectional association between urinary sodium and circulating lipid levels.

Our findings for the relationship between urinary sodium and circulating HDL-C levels are comparable to previous publications. A cross-sectional study involving 223 Chilean individuals (6.9–65.0 years old) indicated an inverse correlation between urinary sodium and HDL-C (*r* = −0.2093, *p* = 0.0018), but it was not significant after adjusting for age, gender, and BMI (23). González and colleagues included 490 patients with mild essential hypertension (144 ± 9/94 ± 9 mmHg, 49.5 ± 13.9 years), and they found significantly low HDL-C levels in the high urinary sodium group (24). Similarly, another cross-sectional study with a larger sample size (1,738 boys aged 10–18 years) also demonstrated a reverse association between higher urinary sodium excretion to urinary specific gravity ratio and lower HDL-C levels (*p* = 0.033) (25). However, the observational studies above failed to assess the causal relation due to the study design. Consistent with a previous MR study with a smaller sample size (*n* = 187,167 vs. ours *n* = 403,943) (26), we demonstrated the causal effect of higher urinary level on lower HDL-C levels. Although the further discovery MVMR analysis indicated a direct causal effect, this effect failed to hold in the replication analysis. It is essential to emphasize that our study is an MR study, designed to offer genetic evidence of potential causal relationships. Although our study hints at a weak causal link between increased urinary sodium and reduced circulating HDL-C levels, caution should be exercised when interpreting this finding since it only suggests a possible causal connection, and further research is needed to validate these results.

The pathophysiology of the association between sodium intake and HDL-C has not been fully elucidated. Emerging evidence suggests that the kidney plays a pivotal role in lipid metabolism, particularly concerning HDL-C. This involvement includes the tubular handling of filtered HDL-C apolipoprotein constituents through the cubilin–megalin–amnionless system (27). Krikken and colleagues proposed a hypothesis that reduced glomerular filtration of HDL-C apolipoproteins contributes to HDL-C catabolism although their findings, indicating that short-term dietary sodium restriction would decrease HDL-C, contradict our results (28). Furthermore, urinary sodium excretion has been positively linked to insulin resistance (29), which can lead to abnormalities in HDL-C levels. In this context, hyperinsulinemia resulting from insulin resistance can promote triglyceride contents in HDL-C particles by enhancing cholesteryl ester transferase activity. This hyperinsulinemia is also a critical factor in reducing plasma HDL-C levels (30). Taken together with our findings, these results suggest that urinary sodium excretion may indeed have a causal impact on HDL-C levels although it may be influenced by other confounding factors. Further studies with larger sample sizes are warranted to provide a more comprehensive understanding of this relationship.

In UVMR, the causal effect of urinary sodium on serum triglycerides was indicated in the discovery analysis. Although it has been reported that higher sodium excretion was related to higher triglyceride levels in several publications (5, 23, 24, 31), these findings were not demonstrated in the replication and further MVMR analysis. Conclusions about such relationships should therefore be treated with caution since they could be confounded by factors like obesity. Reversely, our study has further provided evidence that increased serum triglycerides could elevate urinary sodium in both discovery and replication analyses. In line with previous studies showing that high triglycerides were reported to be associated with high urinary sodium excretions in patients with nephrolithiasis (32, 33), our findings have expanded the causality and supported the direct effects of triglycerides on urinary sodium. In animal experiments, focal and segmental glomerulosclerosis has been found in Dahl salt-sensitive hypertensive rats with a high-salt diet, and Hirano and colleagues found pronounced hypertriglyceridemia in these rats even when they were fed a standard rat chow. Hirano believed that hypertriglyceridemia could be a result of both overproduction and impaired catabolism of very-low-density lipoprotein and triglycerides (34). Furthermore, mineralocorticoid receptor activation has been indicated to promote triglyceride accumulation post-feeding (35). In mice fed with high Na^+^ and high-fat diet, a lower expression of mineralocorticoid receptor was found in the liver (36). Therefore, we hypothesized that the relationship between urinary sodium and triglycerides may be related to the alteration of mineralocorticoid receptors.

There are some strengths in our study. Firstly, compared with a previous MR study (26), a GWAS with a larger sample size was included, and the potential reverse causality between urinary sodium and lipid levels was evaluated. Secondly, we conducted both discovery and replication analysis using samples from the UKB and the Global Lipids Genetics Consortium, which guaranteed the robustness of the results. Thirdly, we additionally applied several rigorous MR methods to assess causality throughout the analysis. However, we acknowledged some limitations. Firstly, the generalizability of our findings may be limited because only individuals with European ancestry were included. Secondly, the sample for urinary sodium and lipid levels is partially overlapped in the UKB part. Although the two-sample MR can still be applied in this situation (32), it may bring the winner’s curse bias, which refers to the phenomenon where the initial results of an association often appear to be exaggerated, deviating significantly from the null hypothesis, while subsequent replication studies tend to yield more conservative estimates (37). Third, although we used MVMR to estimate the association between urinary sodium and the three lipids and the other pleiotropy-resistant MR methods provided consistent results, there remains a potential residual bias due to pleiotropic associations among the lipid variants.

## Conclusion

The major finding is that our study provided genetic evidence that increased urinary sodium may have weak causal effects on decreased circulating HDL-C levels. Furthermore, genetically higher triglyceride levels may have independent causal effects on increased urinary sodium excretion. Moreover, reducing sodium intake may be beneficial for lipid regulation, especially HDL-C. Further interventional studies are warranted to confirm these results.

## Data availability statement

The original contributions presented in the study are included in the article/**Supplementary Material**. Further inquiries can be directed to the corresponding author.

## Author contributions

Conception and design: CY, PJ, and ZJ. Acquisition of data: CY and ZJ. Analysis and interpretation of data: PJ, ZJ, and XW. Drafting of the manuscript: CY, PJ, and ZJ. Critical revision of the manuscript for important intellectual content: XW. Funding acquisition: XW. Administrative, technical, or material support: ZJ and XW. Supervision: XW. All authors contributed to the article and approved the submitted version.
