# Supplementary material for: Association between urinary sodium and circulating lipid levels: a Mendelian randomization study
Source: Front Endocrinol (Lausanne). 2023 Nov 29;14:1189473. doi: 10.3389/fendo.2023.1189473 (PMC10716694; doi:10.3389/fendo.2023.1189473)
Supplement: Supplementary file 1 [file DataSheet_1.docx]

**Supplementary Table S1:** The characteristics of GWAS summary statistics used in the analyses.

GWAS, genome-wide association study; SD, standard deviation; UKB, UK biobank; GLGC, Global Lipids Genetics Consortium; LDL-C: low-density lipoprotein cholesterol; HDL-C: high-density lipoprotein cholesterol

| **Items** | **Subitems** | | **Population** | **Trait or case definition** | **Adjusted covariates** | **PMID** | **Units** |
| --- | --- | --- | --- | --- | --- | --- | --- |
| Urinary sodium | / | | 446,237 European individuals from UK Biobank (UKB) | From urinary sample and measured by the ion selective electrode method (potentiometric method) using Beckman Coulter AU5400, UK Ltd | sex, age | 31409800 | SD |
|  |  | |  |  |  |  |  |
| ***Lipid traits from UKB*** | |  |  |  |  |  |  |
| Circulating lipid level | triglyceride | | European: 441,016 | Details on sample handling and assays used have been previously described on the website (https://biobank.ndph.ox.ac.uk/showcase/showcase/docs/serum_biochemistry.pdf) | age, sex, and genotyping chip array | 32203549 | SD |
|  |  | |  |  |  |  |  |
|  | HDL-C | | European: 403,943 |  |  |  | SD |
|  | LDL-C | | European: 440,546 |  |  |  | SD |
| ***Lipid traits from GLGC*** | |  |  |  |  |  |  |
| Circulating lipid level | triglyceride | | European: 177,861 | Blood lipid levels were typically measured after > 8 hours of fasting. Individuals known to be on lipid-lowering medication were excluded when possible. LDL-C levels were directly measured in 10 studies and estimated using the Friedewald formula in the remaining studies of the GWAS included. | age, age^2^, and sex | 24097068 | SD |
|  | HDL-C | | European: 187,167 |  |  |  |  |
|  | LDL-C | | European: 173,082 |  |  |  |  |

Supplementary table S2 Proportion of variance explained and F statistics for urinary sodium on circulating lipids levels

SNP, single nucleotide polymorphism; LDL-C: low-density lipoprotein cholesterol; HDL-C: high-density lipoprotein cholesterol

| Outcomes | N-SNPs | rsq [%] | sample size | Calculated F-statistics |
| --- | --- | --- | --- | --- |
| ***Discovery*** | | | | |
| LDL-C | 38 | 0.38 | 446237 | 45 |
| HDL-C | 32 | 0.33 | 446237 | 46 |
| Triglycerides | 36 | 0.37 | 446237 | 46 |
| ***Replication*** | | | | |
| LDL-C | 26 | 0.27 | 446237 | 46 |
| HDL-C | 26 | 0.27 | 446237 | 46 |
| Triglycerides | 20 | 0.20 | 446237 | 45 |

The proportion of variance in the exposure explained by the genetic variants (rsq), was calculated using the TwoSampleMR R functions get_r_from_pn ().

The F statistics related to the proportion of variance in the exposure explained by the genetic variants(R2), sample size(N) and number of instruments(K) and was calculated by the formula F = ((N-K-1)/K ) ( R2/(1-R2)).

Supplementary table S3 Mendelian randomization study results of urinary sodium on lipid traits in discovery analysis.

SNP, single nucleotide polymorphism; LDL-C: low-density lipoprotein cholesterol; HDL-C: high-density lipoprotein cholesterol; IVW, inverse variance weighted; SD, standard deviation; LCI, lower confidence interval; UCI, upper confidence interval; Qhet, heterogeneities by Cochran Q statistic; rsq, the proportion of variance in the exposure explained by the genetic variants

| Outcomes | N-SNPs | rsq [%] | MR Methods | Beta | 95%LCI | 95%UCI | pval | Qhet (pval) | Egger_intercept (pval) |
| --- | --- | --- | --- | --- | --- | --- | --- | --- | --- |
| LDL-C | 38 | 0.38 | IVW | -0.047 | -0.153 | 0.059 | 0.380 | 5.38E-03 | / |
|  |  |  | MR Egger | -0.332 | -0.815 | 0.150 | 0.186 | / | 0.244 |
|  |  |  | Weighted median | -0.047 | -0.174 | 0.079 | 0.464 | / | / |
|  |  |  | Weighted mode | 0.001 | -0.275 | 0.278 | 0.992 | / | / |
| HDL-C | 32 | 0.33 | IVW | -0.316 | -0.431 | -0.201 | 7.25E-08 | 1.18E-04 | / |
|  |  |  | MR Egger | -0.128 | -0.574 | 0.318 | 0.579 | / | 0.397 |
|  |  |  | Weighted median | -0.336 | -0.462 | -0.209 | 1.91E-07 | / | / |
|  |  |  | Weighted mode | -0.410 | -0.707 | -0.113 | 0.011 | / | / |
| Triglyceride | 36 | 0.37 | IVW | 0.218 | 0.034 | 0.402 | 0.020 | 5.03E-25 | / |
|  |  |  | MR Egger | 0.130 | -0.595 | 0.855 | 0.727 | / | 0.808 |
|  |  |  | Weighted median | 0.305 | 0.173 | 0.438 | 6.36E-06 | / | / |
|  |  |  | Weighted mode | 0.327 | 0.114 | 0.540 | 0.005 | / | / |

Supplementary table S4 Mendelian randomization study results of urinary sodium on lipid traits in replication analysis.

SNP, single nucleotide polymorphism; LDL-C: low-density lipoprotein cholesterol; HDL-C: high-density lipoprotein cholesterol; IVW, inverse variance weighted; SD, standard deviation; LCI, lower confidence interval; UCI, upper confidence interval; Qhet, heterogeneities by Cochran Q statistic; rsq, the proportion of variance in the exposure explained by the genetic variants

| Outcomes | N-SNPs | rsq [%] | MR Methods | Beta | 95%LCI | 95%UCI | pval | Qhet (pval) | Egger_intercept (pval) |
| --- | --- | --- | --- | --- | --- | --- | --- | --- | --- |
| LDL-C | 26 | 0.27 | IVW | 0.125 | -0.123 | 0.373 | 0.324 | 0.157 | / |
|  |  |  | MR Egger | -0.904 | -2.117 | 0.310 | 0.157 | / | 0.103 |
|  |  |  | Weighted median | 0.056 | -0.275 | 0.387 | 0.741 | / | / |
|  |  |  | Weighted mode | 0.019 | -0.493 | 0.532 | 0.942 | / | / |
| HDL-C | 26 | 0.27 | IVW | -0.371 | -0.598 | -0.144 | 0.001 | 0.097 | / |
|  |  |  | MR Egger | -0.750 | -1.899 | 0.398 | 0.213 | / | 0.515 |
|  |  |  | Weighted median | -0.286 | -0.593 | 0.020 | 0.067 | / | / |
|  |  |  | Weighted mode | -0.285 | -0.907 | 0.337 | 0.377 | / | / |
| Triglyceride | 20 | 0.20 | IVW | 0.239 | -0.011 | 0.489 | 0.061 | 0.266 | / |
|  |  |  | MR Egger | 0.506 | -0.766 | 1.778 | 0.446 | / | 0.680 |
|  |  |  | Weighted median | 0.443 | 0.105 | 0.782 | 0.010 | / | / |
|  |  |  | Weighted mode | 0.506 | -0.062 | 1.074 | 0.097 | / | / |

Supplementary table S5 Mendelian randomization study results of lipid traits on urinary sodium in discovery analysis.

SNP, single nucleotide polymorphism; LDL-C: low-density lipoprotein cholesterol; HDL-C: high-density lipoprotein cholesterol; IVW, inverse variance weighted; SD, standard deviation; LCI, lower confidence interval; UCI, upper confidence interval; Qhet, heterogeneities by Cochran Q statistic; rsq, the proportion of variance in the exposure explained by the genetic variants

| Exposures | N-SNPs | rsq [%] | MR Methods | Beta | 95%LCI | 95%UCI | pval | Qhet (pval) | Egger_intercept (pval) |
| --- | --- | --- | --- | --- | --- | --- | --- | --- | --- |
| LDL-C | 175 | 2.54 | IVW | -0.005 | -0.014 | 0.004 | 0.299 | 1.31E-12 | / |
|  |  |  | MR Egger | -0.018 | -0.031 | -0.004 | 0.011 | / | 0.012 |
|  |  |  | Weighted median | -0.014 | -0.027 | -0.002 | 0.026 | / | / |
|  |  |  | Weighted mode | -0.015 | -0.024 | -0.005 | 0.004 | / | / |
| HDL-C | 434 | 6.16 | IVW | -0.010 | -0.018 | -0.003 | 0.008 | 4.58E-35 | / |
|  |  |  | MR Egger | 0.015 | 0.003 | 0.026 | 0.012 | / | 2.35E-08 |
|  |  |  | Weighted median | 0.003 | -0.007 | 0.013 | 0.564 | / | / |
|  |  |  | Weighted mode | 0.006 | -0.003 | 0.014 | 0.175 | / | / |
| Triglyceride | 374 | 4.72 | IVW | 0.030 | 0.020 | 0.039 | 2.12E-10 | 1.08E-27 | / |
|  |  |  | MR Egger | 0.011 | -0.004 | 0.027 | 0.141 | / | 0.004 |
|  |  |  | Weighted median | 0.019 | 0.006 | 0.031 | 0.003 | / | / |
|  |  |  | Weighted mode | 0.017 | 0.002 | 0.032 | 0.026 | / | / |

Supplementary table S6 Mendelian randomization study results of lipid traits on urinary sodium in replication analysis.

SNP, single nucleotide polymorphism; LDL-C: low-density lipoprotein cholesterol; HDL-C: high-density lipoprotein cholesterol; IVW, inverse variance weighted; SD, standard deviation; LCI, lower confidence interval; UCI, upper confidence interval; Qhet, heterogeneities by Cochran Q statistic; rsq, the proportion of variance in the exposure explained by the genetic variants

| Exposures | N-SNPs | rsq [%] | MR Methods | Beta | 95%LCI | 95%UCI | pval | Qhet (pval) | Egger_intercept (pval) |
| --- | --- | --- | --- | --- | --- | --- | --- | --- | --- |
| LDL-C | 93 | 2.27 | IVW | -0.006 | -0.014 | 0.001 | 0.107 | 9.37E-10 | / |
|  |  |  | MR Egger | -0.009 | -0.020 | 0.003 | 0.138 | / | 0.559 |
|  |  |  | Weighted median | -0.003 | -0.012 | 0.006 | 0.487 | / | / |
|  |  |  | Weighted mode | -0.009 | -0.016 | -0.001 | 0.026 | / | / |
| HDL-C | 116 | 2.51 | IVW | -0.001 | -0.009 | 0.007 | 0.869 | 9.28E-08 | / |
|  |  |  | MR Egger | 0.008 | -0.006 | 0.023 | 0.263 | / | 0.154 |
|  |  |  | Weighted median | -0.001 | -0.012 | 0.009 | 0.790 | / | / |
|  |  |  | Weighted mode | 0.002 | -0.008 | 0.011 | 0.765 | / | / |
| Triglyceride | 65 | 4.72 | IVW | 0.014 | 0.002 | 0.025 | 0.021 | 1.33E-07 | / |
|  |  |  | MR Egger | -0.006 | -0.025 | 0.012 | 0.505 | / | 0.009 |
|  |  |  | Weighted median | 0.001 | -0.011 | 0.014 | 0.824 | / | / |
|  |  |  | Weighted mode | 0.004 | -0.009 | 0.017 | 0.583 | / | / |

Supplementary Table S7 STROBE-MR checklist of recommended items to address in reports of Mendelian randomization studies^[[1]](#endnote-1)^

| **Item No.** | **Section** |  | **Checklist item** | **Page No.** | **Relevant text from manuscript** |
| --- | --- | --- | --- | --- | --- |
| 1 | **TITLE and ABSTRACT** |  | Indicate Mendelian randomization (MR) as the study’s design in the title and/or the abstract if that is a main purpose of the study | **1-2** | **Line 1-59** |
|  | **INTRODUCTION** |  |  |  |  |
| 2 | **Background** |  | Explain the scientific background and rationale for the reported study. What is the exposure? Is a potential causal relationship between exposure and outcome plausible? Justify why MR is a helpful method to address the study question | **3** | **Line 99-112** |
| 3 | **Objectives** |  | State specific objectives clearly, including pre-specified causal hypotheses (if any). State that MR is a method that, under specific assumptions, intends to estimate causal effects | **3** | **Line 124-126** |
|  | **METHODS** |  |  |  |  |
| 4 | **Study design and data sources** |  | Present key elements of the study design early in the article. Consider including a table listing sources of data for all phases of the study. For each data source contributing to the analysis, describe the following: | **3-5** | **Line 129-208** |
|  |  | a) | Setting: Describe the study design and the underlying population, if possible. Describe the setting, locations, and relevant dates, including periods of recruitment, exposure, follow-up, and data collection, when available. | **3** | **Line 129-137** |
|  |  | b) | Participants: Give the eligibility criteria, and the sources and methods of selection of participants. Report the sample size, and whether any power or sample size calculations were carried out prior to the main analysis | **4** | **Line 145-178** |
|  |  | c) | Describe measurement, quality control and selection of genetic variants | **5** | **Line 189-208** |
|  |  | d) | For each exposure, outcome, and other relevant variables, describe methods of assessment and diagnostic criteria for diseases | **4** | **Line 145-178** |
|  |  | e) | Provide details of ethics committee approval and participant informed consent, if relevant | **9** | **Line 396-398** |
| 5 | **Assumptions** |  | Explicitly state the three core IV assumptions for the main analysis (relevance, independence and exclusion restriction) as well assumptions for any additional or sensitivity analysis | **5** | **Line 189-208** |
| 6 | **Statistical methods: main analysis** |  | Describe statistical methods and statistics used | **4-5** | **Line 170-187** |
|  |  | a) | Describe how quantitative variables were handled in the analyses (i.e., scale, units, model) | **5** | **Line 203-208**  **Supplementary table 1** |
|  |  | b) | Describe how genetic variants were handled in the analyses and, if applicable, how their weights were selected | **4-5** | **Line 170-187** |
|  |  | c) | Describe the MR estimator (e.g. two-stage least squares, Wald ratio) and related statistics. Detail the included covariates and, in case of two-sample MR, whether the same covariate set was used for adjustment in the two samples | **4-5** | **Line 170-187** |
|  |  | d) | Explain how missing data were addressed | **4** | **Line 145-178** |
|  |  | e) | If applicable, indicate how multiple testing was addressed | **N/A** | **N/A** |
| 7 | **Assessment of assumptions** |  | Describe any methods or prior knowledge used to assess the assumptions or justify their validity | **5** | **Line 189-208** |
| 8 | **Sensitivity analyses and additional analyses** |  | Describe any sensitivity analyses or additional analyses performed (e.g. comparison of effect estimates from different approaches, independent replication, bias analytic techniques, validation of instruments, simulations) | **4** | **Line 174-184** |
| 9 | **Software and pre-registration** |  |  |  |  |
|  |  | a) | Name statistical software and package(s), including version and settings used | **5** | **Line 203-208** |
|  |  | b) | State whether the study protocol and details were pre-registered (as well as when and where) | **N/A** | **N/A** |
|  | **RESULTS** |  |  |  |  |
| 10 | **Descriptive data** |  |  |  |  |
|  |  | a) | Report the numbers of individuals at each stage of included studies and reasons for exclusion. Consider use of a flow diagram | **5-6** | **Line 214-216**  **Line 258-260**  **Supplementary Fig 1 & 2** |
|  |  | b) | Report summary statistics for phenotypic exposure(s), outcome(s), and other relevant variables (e.g. means, SDs, proportions) | **N/A** | **Supplementary table 3-6** |
|  |  | c) | If the data sources include meta-analyses of previous studies, provide the assessments of heterogeneity across these studies | **N/A** | **N/A** |
|  |  | d) | For two-sample MR:  i. Provide justification of the similarity of the genetic variant-exposure associations between the exposure and outcome samples  ii. Provide information on the number of individuals who overlap between the exposure and outcome studies | **4** | **Line 145-178Supplementary table 1** |
| 11 | **Main results** |  |  |  |  |
|  |  | a) | Report the associations between genetic variant and exposure, and between genetic variant and outcome, preferably on an interpretable scale | **5-6** | **Line 214-275**  **Supplementary table 3-6** |
|  |  | b) | Report MR estimates of the relationship between exposure and outcome, and the measures of uncertainty from the MR analysis, on an interpretable scale, such as odds ratio or relative risk per SD difference | **5-6** | **Line 214-275**  **Supplementary table 3-6** |
|  |  | c) | If relevant, consider translating estimates of relative risk into absolute risk for a meaningful time period | **N/A** | **N/A** |
|  |  | d) | Consider plots to visualize results (e.g. forest plot, scatterplot of associations between genetic variants and outcome versus between genetic variants and exposure) | **N/A** | **fig 1-4** |
| 12 | **Assessment of assumptions** |  |  |  |  |
|  |  | a) | Report the assessment of the validity of the assumptions | **5-6** | **Line 214-216**  **Line 258-260** |
|  |  | b) | Report any additional statistics (e.g., assessments of heterogeneity across genetic variants, such as *I*^2^ , Q statistic or E-value) | **5-6** | **Line 243-245**  **Supplementary table 2-6** |
| 13 | **Sensitivity analyses and additional analyses** |  |  |  |  |
|  |  | a) | Report any sensitivity analyses to assess the robustness of the main results to violations of the assumptions | **N/A** | **Supplementary table 3-6** |
|  |  | b) | Report results from other sensitivity analyses or additional analyses | **N/A** | **Supplementary table 3-6** |
|  |  | c) | Report any assessment of direction of causal relationship (e.g., bidirectional MR) | **5-6** | **Line 223-275**  **Supplementary table 3-6** |
|  |  | d) | When relevant, report and compare with estimates from non-MR analyses | **N/A** | **N/A** |
|  |  | **e)** | Consider additional plots to visualize results (e.g., leave-one-out analyses) | **N/A** | **Fig 1-4** |
|  | **DISCUSSION** |  |  |  |  |
| 14 | **Key results** |  | Summarize key results with reference to study objectives | **7** | **Line 297-302** |
| 15 | **Limitations** |  | Discuss limitations of the study, taking into account the validity of the IV assumptions, other sources of potential bias, and imprecision. Discuss both direction and magnitude of any potential bias and any efforts to address them | **8** | **Line 348-359** |
| 16 | **Interpretation** |  |  |  |  |
|  |  | a) | Meaning: Give a cautious overall interpretation of results in the context of their limitations and in comparison with other studies | **8** | **Line 353-359** |
|  |  | b) | Mechanism: Discuss underlying biological mechanisms that could drive a potential causal relationship between the investigated exposure and the outcome, and whether the gene-environment equivalence assumption is reasonable. Use causal language carefully, clarifying that IV estimates may provide causal effects only under certain assumptions | **7-8** | **Line 317-327**  **Line 342-346** |
|  |  | c) | Clinical relevance: Discuss whether the results have clinical or public policy relevance, and to what extent they inform effect sizes of possible interventions | **N/A** | **N/A** |
| 17 | **Generalizability** |  | Discuss the generalizability of the study results (a) to other populations, (b) across other exposure periods/timings, and (c) across other levels of exposure | **8** | **Line 353-359** |
|  | **OTHER INFORMATION** |  |  |  |  |
| 18 | **Funding** |  | Describe sources of funding and the role of funders in the present study and, if applicable, sources of funding for the databases and original study or studies on which the present study is based | **9** | **Line 379-380** |
| 19 | **Data and data sharing** |  | Provide the data used to perform all analyses or report where and how the data can be accessed, and reference these sources in the article. Provide the statistical code needed to reproduce the results in the article, or report whether the code is publicly accessible and if so, where | **9** | **Line 401-407** |
| 20 | **Conflicts of Interest** |  | All authors should declare all potential conflicts of interest | **9** | **Line 382-383** |

1. Skrivankova VW, Richmond RC, Woolf BAR, Yarmolinsky J, Davies NM, Swanson SA, VanderWeele TJ, Higgins JPT, Timpson NJ, Dimou N, Langenberg C, Golub RM, Loder EW, Gallo V, Tybjaerg-Hansen A, Davey Smith G, Egger M, Richards JB. Strengthening the Reporting of Observational Studies in Epidemiology Using Mendelian Randomization: The STROBE-MR Statement. JAMA. 2021 Oct 26;326(16):1614-1621. doi: 10.1001/jama.2021.18236. PMID: 34698778.

   Supplementary fig 1 Flowgraph for Mendelian randomization study urinary sodium on lipid traits

   Supplementary fig 2 Flowgraph for Mendelian randomization study lipid traits on urinary sodium


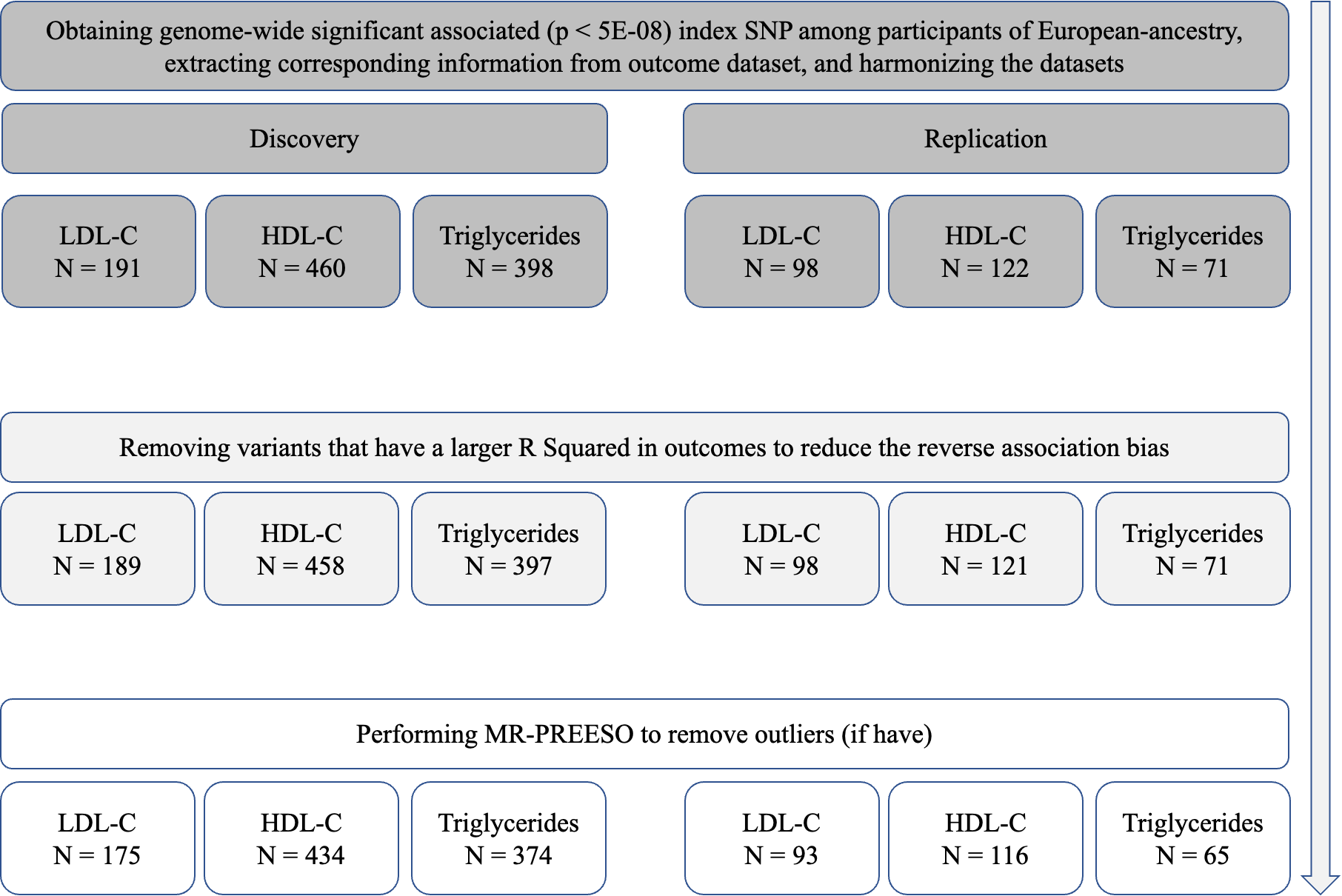
 [↑](#endnote-ref-1)
